# Esophageal Infusion of Menthol Does Not Affect Esophageal Motility in Patients with Gastroesophageal Reflux Disease

**DOI:** 10.1007/s00455-023-10617-7

**Published:** 2023-09-20

**Authors:** Peter Bánovčin, Peter Lipták, Diana Vážanová, Jakub Hoferica, Miloš Tatár, Martin Ďuriček

**Affiliations:** 1 Clinic of Internal Medicine - Gastroenterology, Jessenius Faculty of Medicine in Martin (JFM CU), Comenius University in Bratislava, Kollárova 2, 03659 Martin, Slovakia; 2 Department of Pathological Physiology, Jessenius Faculty of Medicine in Martin (JFM CU), Comenius University in Bratislava, Malá Hora 4C, 036 01 Martin, Slovakia

**Keywords:** Menthol, Gastroesophageal reflux disease, Esophageal motility, High resolution manometry

## Abstract

Menthol is thought to trigger gastroesophageal reflux disease (GERD) symptoms by influencing esophageal peristalsis and lower esophageal sphincter (LES) function. We evaluated the effect of esophageal menthol infusion on esophageal motility and the LES in healthy volunteers and in patients with GERD. High resolution manometry (HRM) catheter with attached thin tube for menthol infusion was placed transnasally. Protocol which included baseline recording, 16 water swallows (5 ml, 10 ml, and 15 ml) and the multiple rapid swallows was performed before and after esophageal infusion of menthol (3 mM, 20 min, 8 ml/min). We evaluated the effect of this infusion on the HRM parameters of esophageal peristalsis (distal contractile integral, distal latency, contractile front velocity) and the lower esophageal sphincter (LES) barrier function (integrated relaxation pressure and the inspiratory augmentation of the LES). Simultaneously we evaluated the quality and intensity of the symptoms during the menthol infusion. Esophageal infusion of menthol did not appreciably affect HRM measurements characterizing esophageal peristalsis and LES pressure in healthy subjects (*N* = 13) or GERD patients (*N* = 11). The magnitude of the distal contractile integral (5 ml) was changed neither in the healthy volunteers’ group, (735 ± 127 vs. 814 ± 117 mmHg, *p* = 0.5), nor in the GERD patients (295 ± 78 vs. 338 ± 96 mmHg, *p* = 0.99). In healthy volunteers menthol did not change the inspiratory augmentation of the LES (8.67 ± 1.09 vs. 7.69 ± 0.96 mmHg, *p* = 0.15) and neither did for GERD patients (8.8 ± 1.18 vs. 8.22 ± 0.91 mmHg, *p* = 0.43). We observed no significant difference in any HRM parameter following menthol infusion, except for distal latency in 10 ml swallows. By contrast, menthol infusion induced significantly more intense discomfort in GERD patient than in healthy volunteers. Our results suggest no significant temporal effect of menthol on the esophageal motility or LES function, neither in healthy volunteers, nor in GERD. Arguably, other mechanisms are responsible for menthol-related heartburn.

**Supplementary Information:**

The online version contains supplementary material available at 10.1007/s00455-023-10617-7.

## Introduction

Menthol is common food additive that has been ascribed properties affecting the function of the gastrointestinal tract. Initial studies reported its involvement in the gastrointestinal neuromotor function, including smooth muscle relaxation [1–3], followed by the demonstration of anti-inflammatory properties [4]. Recently, however, menthol gained attention due to the effect on the gastrointestinal visceral sensation [5] where we demonstrated its ability to provoke heartburn in patients with gastroesophageal reflux disease (GERD) [6]. Indeed, menthol/peppermint oil is in everyday clinical practice often advised to be avoided in GERD patients as it is believed to trigger GERD symptoms. On the other hand, remedies that are used to treat GERD (e.g., alginate) use menthol as a flavorant.

Available evidence indicates that menthol acts via calcium channel blockade when affecting the gastrointestinal motor function while the effect on visceral sensation is mediated through the cold receptor–transient receptor potential cation channel subfamily M member 8 (TRPM8) [7].

Recommendations suggesting avoiding menthol in GERD are most probably based on the mechanistic studies reporting that menthol decreased lower esophageal sphincter (LES) pressure and increased the likelihood of reflux [8]. These were conducted before the high resolution manometry era and therefore lacked the demonstration of the comprehensive effect of menthol on the esophageal motility. The observation that menthol induced heartburn in GERD patients requires ruling out the possibility of affecting the esophageal motility. Therefore, we aimed to explore the effect of menthol infusion on esophageal peristalsis and barrier function of the lower esophageal sphincter (LES) in both healthy volunteers and in GERD patients.

## Methods

This was a prospective single center study. The protocol of the study was approved by the decision of the Ethical Committee of Jessenius Faculty of Medicine, Comenius University with the number EK 1604/2014 in December 2014. The protocol conforms to the ethical guidelines of the 1975 Declaration of Helsinki as reflected in a priori approval by the institution’s human research committee. Written informed consent was obtained from each subject included in the study.

Patients with GERD and chronic heartburn were prospectively enrolled. A careful interview was conducted to assess symptoms of GERD. Inclusion criteria were a chronic (> 6 months) burning sensation behind the sternum (heartburn), with frequency of at least one day per week. Exclusion criteria were age less than 18 years, infection of the upper or lower airways in last 6 weeks, smoking, alcohol consumption > 40 g/day, history of thoracic or abdominal surgery, active malignancy, inflammatory bowel disease, significant renal, cardiac, and interstitial lung disease. Patients on proton pump inhibitor treatment were instructed to discontinue the treatment 4 weeks before the menthol infusion. None of the patients were taking medication affecting esophageal motility or sensitivity (neuromodulators, opioids, prokinetics). Of these patients, 6 had confirmed GERD by esophageal pH/impedance study performed off PPI (proton pump inhibitor) (positive DeMeester score > 14.72). One patient had negative pH/impedance recording and the remaining patients did not receive pH/impedance study and were enrolled based on symptoms only. Healthy volunteers (HV) that have neither any gastrointestinal disease diagnosed, nor they reported any gastrointestinal symptoms (including heartburn) served as a control group. None of the healthy volunteers takes PPI therapy.

Esophageal motility studies were performed as described in our previous study [6]. HRM was performed after overnight fasting. 4.2 mm solid state catheter with 36 circumferential channels spaced in 1 cm intervals (Given Imaging) was used. After the calibration, the catheter was inserted into a disposable sheath (Given Imaging), and a silicone infusion tube (I.D. = 0.8 mm) for menthol infusion was attached to the sheath. The manometry/infusion assembly was placed transnasally and positioned to record esophageal pressure from the hypopharynx to the stomach while the opening of the silicone tube was positioned at approximately 10 cm above the upper margin of the LES determined by HRM. Studies were performed in the sitting position.

The pre-menthol motility study included the measurement of the esophageal “baseline,” followed by 10 × 5, 3 × 10, and 3 × 15 ml liquid swallows and finally the multiple rapid swallows. Subsequently the infusion silicone tube was connected to a peristaltic pump (PCD 21 M, Kouril, Czech Republic). The infusion rate of the (–)-menthol solution was 8 ml/min resulting in infusion of total volume of 160 ml for 20 min. 3 g of (–)-menthol was dissolved in 3 ml of ethanol to stock solution concentration of 3.2 M. 188 µl of the stock solution was diluted in 200 ml of water to the final concentration of 3 mM. although the EC_50_ of human TRPM8 has not been reported, the EC_50_ of menthol on TRPM8 in rodent species is reported to be 15–75 μM and is considered conserved among species [9]. The menthol concentration 3 mM is 100–300 times the EC_50_ of menthol on TRPM8. based on these studies and the resistance of esophageal epithelium. The rate 8 ml/min. was based on thorough studies focusing on the mechanisms of central sensitization and esophageal pain [10] and was also used in previous studies [6, 11]. The positioning of the outlet was chosen not to influence the proximal esophagus (and its striated muscle) and also the trigeminal nerve with the abundance of TRPM8 receptors. Within 1 min after the termination of menthol infusion the same motility study was performed. The dose and concentration of menthol infusion was based on the assumptive EC_50_ of menthol on TRPM8 receptor with regard to the resistance of esophageal epithelium and is in detail discussed in our previous study [6]. Also, subsequent motility studies performed by other investigators [11] used the same concentration.

Data obtained from the HRM studies were analyzed using ManoView software (Given Imaging) and standard manometric parameters according to the Chicago classification v3.0 were obtained. These included the 4 s integrated relaxation pressure (IRP), distal contractile integral (DCI), and distal latency (DL). Despite not directly included in the v3.0 Chicago classification, the contractile front velocity (CFV) was also evaluated to obtain a comprehensive view on esophageal motility. Parameters were separately analyzed for 5, 10, and 15 ml swallows and for the multiple rapid swallow test. Inspiratory augmentation of the lower esophageal sphincter (mmHg) calculation was performed as described elsewhere [12] as this parameter showed consistency and low variability during the HRM study. HRM studies were evaluated by investigators experienced in the analysis of the esophageal motility (PB, MD), that were blinded to the results of pH/impedance studies.

Importantly, symptoms were evaluated during the menthol infusion as described in our previous study [6]. Briefly, before the infusion the qualified assistant explained to the subject how to rate the discomfort/pain on the visual analog scale (VAS; 1–10, 1 = no sensation, 10 = unbearable discomfort/pain). At 1 min after the infusion was turned on, the assistant asked whether the subject perceived any sensation and to describe the quality of the sensation. In 2-min intervals starting at the second minute the subject was prompted by the assistant to mark the intensity of discomfort/pain and asked whether the quality of the sensation was the same or if it had changed.

### Statistical analysis

All data were presented as mean ± standard error of the mean. Normality of data were checked using Shapiro–Wilk test. Data before and after menthol infusion were compared using paired *T* test or Wilcoxon test. Differences were considered statistically significant at *p* < 0.05. For comparison of the symptom intensity in response to the menthol infusion between the groups the area under the curve (AUC) was calculated AUC = ∑ (VAS_*i*_ + VAS_*i*+1_)/2 for *i* = 1 to 9 where VAS_*i*_ indicates the *i*th VAS as recorded in 2-min intervals.

## Results

Esophageal menthol infusions were performed in 13 healthy subjects (8M/5F), mean age 24.8 [21–31] and 11 patients with chronic heartburn (7M/4F), mean age 31[21–63]. The mean acid exposure time and DeMeester score in the pH positive group of GERD patients was 7.98 ± 0.88 and 30.4 ± 9.99, respectively. Symptom index and symptom association probability (SAP) were available in all pH positive patients and was positive in 5 patients and negative in 1 patient (mean SI 83.38 ± 24.1 and SAP 97.33 ± 4.68).

### Pre-Menthol vs. Post-Menthol Data in Healthy Volunteers and GERD Patients

**Fig. 1:**
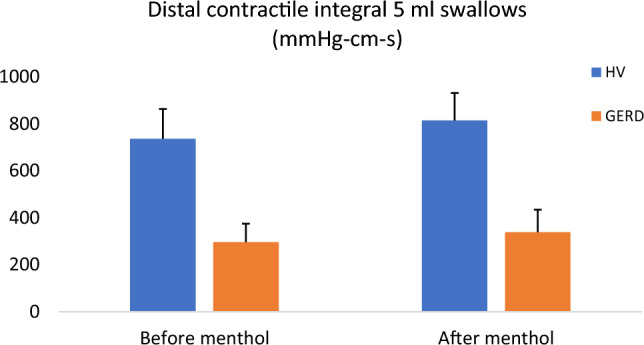
No effect of menthol infusion on the peristaltic vigor. Although the DCI of 5 ml swallows was significantly lower in the HV group compared to GERD (*p* = 0.011), there was no significant difference in the DCI in both HV group and GERD patients following the menthol infusion (*p* = 0.497 and *p* = 0.999, respectively)

**Fig. 2:**
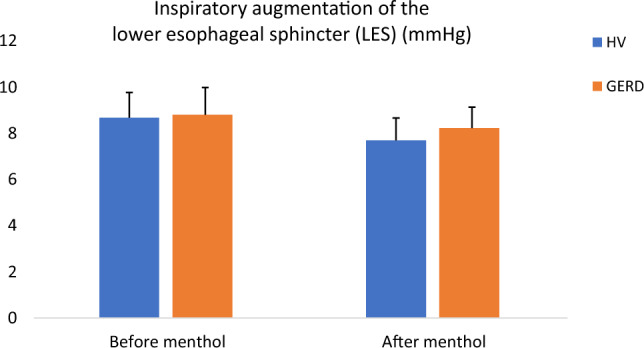
No effect of menthol on the barrier function of the LES. Inspiratory augmentation of the LES shows no significant difference after the menthol infusion in the HV group (*p* = 0.15) and also in the group with GERD patients (*p* = 0.43)

Parameters of esophageal motility and the LES function before and after the menthol infusion are provided in the comprehensive table (Table 1). Not surprisingly, GERD patients had significantly lower peristaltic vigor as evaluated by the DCI compared to the HV group (Fig. 1). Inspiratory augmentation of the LES showed no statistically significant difference between the HV and GERD group (*p* = 0.94) (Fig. 2). It is clearly demonstrated that menthol infusion resulted neither significant effect on the parameters of esophageal peristalsis (Fig. 1), nor the LES function (Fig. 2). The only difference observed was the decrease of the IRP in the multiple rapid swallows (but not standard liquid swallows) in the HV group and the increase in the distal latency (only in 10 ml liquid swallows). Similarly, this increase in the distal latency was detected in the group of GERD patients (both for 10 and 15 ml swallows).

**Table 1:** The difference between parameters of esophageal motility and LES function in the group of healthy volunteers (*n* = 13) and GERD patients (*n* = 11)

	Healthy volunteers			GERD		
	Pre-menthol	Post-menthol	*p* value	Pre-menthol	Post-menthol	*p* value
IRP (mmHg)						
5 ml	2.76 (0.67)	2.08 (0.44)	0.11	4.68 (0.82)	3.25 (0.49)	0.102
10 ml	2.62 (0.75)	1.9 (0.52)	0.216	4.37 (0.78)	3.16 (0.39)	0.24
15 ml	2.3 (0.54)	2.23 (0.55)	0.497	3.72 (0.86)	3 (0.43)	0.638
MRS	1.55 (0.32)	0.68 (0.16)	0.004	3.1 (0.81)	1.98 (0.46)	0.212
Inspiratory LES augmentation	8.67 (1.09)	7.69 (0.96)	0.15	8.8 (1.18)	8.22 (0.91)	0.43
DCI (mmHg cm s)						
5 ml	735 (127)	814 (117)	0.497	295 (78)	338 (96)	0.999
10 ml	986 (156)	943 (164)	0.735	279 (87)	429 (125)	0.083
15 ml	1072 (173)	1117 (180)	0.685	299 (83)	447 (108)	0.24
MRS	1825 (257)	1801 (352)	0.893	450 (133)	635 (180)	0.365
DL (s)						
5 ml	7.11 (0.3)	7 (0.27)	0.591	7.69 (0.61)	7.46 (0.45)	0.964
10 ml	6.79 (0.22)	7.37 (0.33)	0.034	6.79 (0.39)	7.47 (0.42)	0.046
15 ml	7.09 (0.23)	7.37 (0.25)	0.224	6.61 (0.29)	8.27 (0.78)	0.016
MRS	8.66 (0.32)	7.69 (0.56)	0.104	7.3 (0.27)	7.36 (0.51)	0.899
CFV (cm s)						
5 ml	4.05 (0.3)	3.67 (0.26)	0.048	3.46 (0.21)	3.34 (0.16)	0.239
10 ml	3.91 (0.22)	3.8 (0.39)	0.653	3.85 (0.27)	3.29 (0.17)	0.084
15 ml	3.79 (0.21)	3.53 (0.24)	0.135	3.72 (0.24)	3.1 (0.18)	0.113
MRS	2.88 (0.23)	3.79 (0.66)	0.024	3.01 (0.18)	3.08 (0.25)	0.833

Data are shown as mean and standard error of the mean. Statistical difference between particular parameters before and after the menthol infusion are presented and the difference between pre-menthol parameters in HV vs. GERD patients is shown in the last column

*LES* lower esophageal sphincter, *MRS* multiple rapid swallows, *IRP* integrated relaxation pressure, *DCI* distal contractile integral, *DL* distal latency, *CFV* contractile front velocity, *EGJ-CI* esophagogastric junction contractile integral

Furthermore, we determined the rate of ineffective swallows (% of swallows with DCI < 450 mmHg cm s) in 5, 10, and 15 ml swallows both for HV and GERD patients. In healthy volunteers, the % of ineffective swallows before and after the menthol infusion was 40 ± 9.27 vs. 24.62 ± 7.3 (*p* = 0.17) in 5 ml swallows, 3.85 ± 1.4 vs. 7.69 ± 3.23 (*p* = 0.29) in 10 ml swallows, and 6.15 ± 3.11 vs. 4.62 ± 2.15 (*p* = 0.69) in 15 ml swallows, respectively. In GERD patients, the % of ineffective swallows before and after menthol infusion was 72.73 ± 8.43 vs. 67.27 ± 11.92 (*p* = 0.47) in 5 ml swallows, 22.73 ± 3.59 vs. 18.18 ± 4 (*p* = 0.14) in 10 ml swallows, and 20.91 ± 4.15 vs. 16.36 ± 3.88 (*p* = 0.24) in 15 ml swallows for 5, 10, and 15 ml swallows, respectively. To conclude, menthol infusion led to no significant change of the rate of ineffective swallows both in HV and GERD patients.

As there were 6 patients with GERD confirmed by pH/impedance, we performed subgroup analysis of these patients. Firstly, compared to the remaining GERD patients, there were no significant differences in the parameters of esophageal motility. Secondly, separate analysis of these patients revealed no significant effect of menthol infusion on their motility parameters, apart from DL in 15 ml swallows (see Supplementary Material).

### Symptoms in Response to the Menthol Infusion

**Fig. 3:**
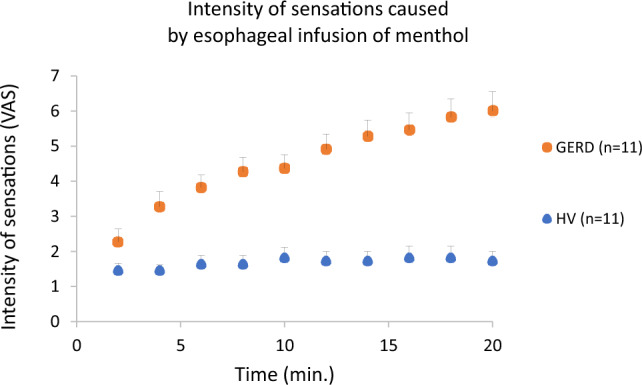
Intensity of esophageal pain/discomfort caused by the menthol infusion. The mean VAS score at the end of the menthol infusion is significantly lower in the group of healthy volunteers (*p* < 0.0001) than the GERD patients

Of the 13 healthy volunteers, 11 had data on their symptoms available. All HV reported cold sensation behind the sternum, which changed into mild heartburn during the course of the infusion in 2 subjects. All 11 GERD patients had data on their symptoms available. All patients with GERD reported heartburn during their menthol infusion. The intensity of discomfort/pain was significantly different between the HV and GERD groups. The mean VAS score (range 0–10) at the end of the menthol infusion (20 min.) in the HV group was 1.73 ± 0.27, while in the GERD group it was 6 ± 0.56 (*p* < 0.0001, unpaired *T* test) (Fig. 3). Importantly, separate analysis of the pH metry positive GERD subgroup and subgroup that did not have pH metry showed insignificant differences (6 ± 0.77 vs. 6 ± 0.89, *p* = 1).

## Discussion

The significance of the results of our study could be summarized as follows: menthol infusion elicited neither the effect on the amplitude, vigor, and integrity of the primary peristalsis nor the barrier function of the LES. No impact on the esophageal peristalsis parameters was observed despite significant differences in the baseline peristaltic activity between the healthy volunteers and the GERD patients. We observed only marginal significance in the increase of the distal latency both in healthy volunteers, and in GERD patients. To the best of our knowledge, this is the first high resolution manometry study evaluating the effect of the menthol infusion in patients with GERD.

The issue of the influence of menthol on the esophageal motility has several aspects. From the clinical point of view, alteration of esophageal motility and LES function that could theoretically lead to more reflux is particularly relevant in the context of dietary recommendations suggesting avoiding menthol and/or peppermint oil in patients with GERD. These were most probably based on the clinicians’ and patients’ observation that menthol induces heartburn, and further supported by older studies delineating the altered barrier function of the LES and triggering reflux. The scientific viewpoint is related to the (potential) ability of intraesophageal menthol to alter esophageal motility as was already demonstrated in other substances, e.g., acid or capsaicin [13, 14]. Proximity of afferent receptors to the esophageal luminal surface can impact sensory perception and consequently, peristaltic vigor [15, 16].

The physiologic effect of peppermint oil/menthol on the gastrointestinal tract involves the impact on both the motor function and the esophageal sensitivity. The motor function was investigated, and the antispasmodic and cramps-alleviating impact of peppermint oil was utilized in the symptomatic treatment of irritable bowel syndrome (IBS), although without significant difference from placebo [17–19]. Available evidence suggests that the capability of menthol to block calcium channels is most probably involved [20]. According to in vitro studies, peppermint oil also antagonizes serotonine-induced contraction and reverses acetylcholine-induced contraction [1]. It was the aim of the studies performed previously to ascertain whether similar effect was possible in the esophagus. Studies with peppermint oil demonstrated the decrease of the LES pressure and the increase of the likelihood of reflux by equating the pressures across esophageal body, LES, and stomach [8]. These results defy ours, however, their study employed conventional manometry (not HRM). Peppermint oil was also demonstrated to decrease esophageal spasms [21]. It is questionable whether the significant increase of distal latency that we observed in 10 and 15 ml swallows reflect the same mechanism that led to the reduction of spasms. Nevertheless, we are cautious about overinterpretation as the significance was neither reproduced for 5 ml swallows, nor for the multiple rapid swallows. As our study included only patients with normal or ineffective motility, the results are not directly comparable.

We observed that following the esophageal menthol infusion there was no significant effect on the esophageal motility parameters, related either to the esophageal body contractility or the LES barrier function. When evaluating the effect of menthol on the barrier function of the LES we consider the inspiratory augmentation of the LES a relevant parameter as it is correlated with the positive pH metry findings [12, 22]. In the group of GERD patients, the inspiratory augmentation of the LES is of nonsignificant decrease (8.8 ± 1.18 vs. 8.22 ± 0.91 mmHg, *p* = 0.43) and so is in healthy volunteers (8.67 ± 1.09 vs. 7.69 ± 0.96 mmHg, *p* = 0.15) (Table 1).

The only difference between the pre- and post-menthol was the decrease of the IRP in the multiple rapid swallows and the increase in the distal latency. We suggest caution in the interpretation of the IRP finding because the pathophysiological relevance of this parameter in terms of GERD has not been demonstrated. Also, the DL result must not be overemphasized, as is difficult to assess outside a distal esophageal spasm patient cohort.

During the menthol infusion, we observed similar symptom pattern in terms of the quality (cold sensation, heartburn) and intensity as was already reported by our group [6]. Menthol infusion induced mostly cold sensation with mild intensity in HV group, however, heartburn was reported in all GERD patients, with significantly higher intensity (Fig. 3). This observation suggests that rather direct stimulation of esophageal sensory afferents (and possibly TRPM8 receptors) than alteration of esophageal motility is responsible for the symptoms.

Investigation of the effect of menthol on the esophageal motor function was also the intention of the recent HRM study, similar to ours in terms of the design and the concentration and duration of menthol infusion used [11]. This study also performed esophageal infusion of the placebo (saline), however, included only healthy volunteers and no patients with GERD of heartburn. This study demonstrated the effect of menthol infusion on the reduction of the basal UES (upper esophageal sphincter) pressure and the frequency of secondary peristalsis induced by rapid air injections. Otherwise, no effect of menthol infusion on the parameters of esophageal peristalsis was shown, which is in accordance with our results. The authors assume that this mild effect of menthol infusion on the esophageal motor function is modulated by the activation of TRPM8 receptors.

Taken together, the effect of menthol on primary esophageal peristalsis and the LES function seems to be mild. Despite that, this does not particularly rule out the possibility of menthol of triggering reflux or extend contact of the refluxate and the esophageal mucosa as impaired esophageal peristalsis is only one of the pathophysiological mechanisms of GERD.

The possibility of menthol to trigger transient lower esophageal sphincter relaxations (TLESRs) has not been investigated yet. This could be a mechanism of relevance as, e.g., postprandial acid infusions can increase the number of TLESRs [23]. Another option, yet still not investigated is the ability of esophageal luminal content (e.g., intraesophageal menthol) to affect the contraction amplitude due to the proximity of afferent (e.g., TRPM8) receptors to the esophageal luminal surface. Although proven for capsaicin [13], our results do not support this concept. What is more, the putative mechanism that would explain the direct role of TRPM8 receptors on the esophageal motor function has not been established.

Our study has limitations. Firstly, the protocol of our study did not include the infusion that would serve as a control. Therefore, despite not substantial, the changes of the esophageal motility (distal latency) carry the eventuality of the placebo effect. This was partially overcome by the fact that neither the healthy volunteers, nor patients with GERD symptoms were aware of the composition of the menthol infusion. Still, the not-interchangable scent of menthol lead the patients to correctly sense that the infusion is indeed the menthol solution. Secondly, we assessed only primary peristalsis and the function of the LES. Thus, one cannot exclude the effect of menthol on secondary peristalsis and the esophageal clearance as its impairment could prolong the contact time between the refluxate and esophageal mucosa. Finally, the number of GERD patients confirmed by pH/impedance is somewhat low and the rest was included based on symptoms One cannot exclude the possibility of, e.g., reflux hypersensitivity in this subset of patients.

## Conclusion

Esophageal menthol infusion seems to have limited impact on esophageal motor function both in patients with GERD and the healthy volunteers. Further studies employing extended high resolution impedance manometry, ideally focusing on the both esophageal motility and UES function including impedance metrics, with various bolus consistencies and the subsequent analysis of the bolus clearance, number of TLESR and/or using pH/impedance after the menthol infusion would provide a comprehensive picture on the effect of menthol on the esophageal motility and aid further to elucidate this issue.

### Supplementary Information

Below is the link to the electronic supplementary material.

Supplementary file1 (DOCX 18 kb)
