# Supplementary material for: Esophageal Infusion of Menthol Does Not Affect Esophageal Motility in Patients with Gastroesophageal Reflux Disease
Source: Dysphagia. 2023 Sep 20;39(3):369–75. doi: 10.1007/s00455-023-10617-7 (PMC11127881; doi:10.1007/s00455-023-10617-7)
Supplement: Supplementary file 1 — Supplementary file1 (DOCX 18 kb) [file 455_2023_10617_MOESM1_ESM.docx]

|  | **Pre-menthol** | | | **Post-menthol** | |
| --- | --- | --- | --- | --- | --- |
| **IRP (mmHg)** | **pH positive GERD patients** | **GERD patients without pH/impedance** | **p value** | **pH positive GERD patients** | **p value** |
| 5 ml | 5,26±1,26 | 4,02±1,07 | 0,5 | 3,35±0,79 | 0,26 |
| 10 ml | 4,67±1,28 | 4,02±0,93 | 0,7 | 3,15±0,69 | 0,32 |
| 15 ml | 4,42±1,34 | 2,88±1,04 | 0,4 | 3,32±0,73 | 0,59 |
| MRS | 3,47±1,34 | 2,66±0,89 | 0,64 | 2,03±0,7 | 0,52 |
| Inspiratory LES augmentation | 8,67±1,86 | 9±1,51 | 0,9 | 7,8±1,45 | 0,53 |
| **DCI** |  |  |  |  |  |
| **(mmHg-cm-s)** |  |  |  |  |  |
| 5 ml | 292±122 | 299±107 | 0,97 | 278±138 | 0,71 |
| 10 ml | 211±107 | 361±147 | 0,42 | 286±141 | 0,1 |
| 15 ml | 246±108 | 361±137 | 0,52 | 391±125 | 0,18 |
| MRS | 232±58 | 711±247 | 0,07 | 580±230 | 0,22 |
| **DL (s)** |  |  |  |  |  |
| 5 ml | 7,23±0,63 | 8,06±1,09 | 0,57 | 6,94±0,61 | 0,77 |
| 10 ml | 6,49±0,61 | 7,09±0,52 | 0,55 | 7,02±0,78 | 0,12 |
| 15 ml | 6,09±0,31 | 7,136±0,4 | 0,08 | 7,49±0,48 | 0,02 |
| MRS | 7,62±0,39 | 6,9±0,33 | 0,26 | 7,5±0,9 | 0,81 |
| **CFV (cm-s)** |  |  |  |  |  |
| 5 ml | 3,17±0,27 | 3,69±0,32 | 0,31 | 3,42±0,26 | 0,3 |
| 10 ml | 3,29±0,3 | 4,41±0,32 | 0,07 | 3,22±0,25 | 0,73 |
| 15 ml | 3,42±0,25 | 4,02±0,41 | 0,26 | 3,1±0,28 | 0,44 |
| MRS | 2,78±0,19 | 3,3±0,31 | 0,21 | 3,2±0,48 | 0,37 |

Supplementary table. HRM parameters in the group of GERD patients. The pre-menthol part shows differences between motility parameters of GERD patients confirmed by pH/impedance (n=6) compared to the patients not receiving pH/impedance (unpaired T-test) (n=5). The post-menthol part shows the difference between motility parameters in the pH positive GERD patients (n=6). All parameters except for 15 ml DL show no statistically significant difference following the menthol infusion. GERD – gastroesophageal reflux disease, HRM – high resolution manometry, DL – distal latency
